# A real-world data study of the effect of co-solvent differences on the clinical safety of melphalan for injection

**DOI:** 10.3389/fmed.2025.1554818

**Published:** 2025-09-26

**Authors:** Guorong Wang, Wenming Chen, Chenwei Zuo, Huang Li, Jianzhou Yan

**Affiliations:** ^1^Department of Hematology, Beijing Medical Research Center for Multiple Myeloma, Beijing Chaoyang Hospital Affiliated to Capital Medical University, Beijing, China; ^2^School of International Pharmaceutical Business, China Pharmaceutical University, Nanjing, China; ^3^Research Center of National Drug Policy and Ecosystem, China Pharmaceutical University, Nanjing, China

**Keywords:** co-solvent, EVOMELA^®^, propylene glycol melphalan, multiple myeloma, autologous stem cell transplantation

## Abstract

**Objective:**

This study aimed to compare the efficacy and safety of myeloablative conditioning with high-dose propylene glycol-free melphalan (PGF-Mel, EVOMELA^®^) versus propylene glycol melphalan (PG-Mel) in Chinese patients with multiple myeloma (MM) undergoing autologous stem cell transplantation (ASCT) in the real world.

**Methods:**

This is a single-center, retrospective study of 107 patients with MM. Patients were divided into two groups based on their high-dose myeloablative conditioning regimen before autologous stem cell transplantation (ASCT): the EVOMELA^®^ group (53 patients) and the PG-Mel group (54 patients). Qualitative data were compared using the two independent samples t-test and two independent samples Mann–Whitney U-test, and quantitative data were compared using the chi-square test. Efficacy and safety parameters were assessed and compared between the two groups.

**Results:**

The median time to myeloablation, the median time to platelet engraftment, and the neutrophil nadir were significantly lower in the EVOMELA^®^ group than in the PG-Mel group (*p* < 0.05). In contrast, the median time to neutrophil engraftment, the lymphocyte nadir, and the number of platelet transfusions did not differ significantly between the two groups (*p* > 0.05). Adverse events, such as ionic disorders (hypokalemia, hypocalcemia, hypophosphatemia), nausea, diarrhea, vomiting, and loss of appetite, are significantly lower in the EVOMELA^®^ group than in the PG-Mel group (*p* < 0.05), and the incidence of pyrexia between the two groups is not statistically different (*p* > 0.05). The average length of a hospitalization stay between the two groups was similar (*p* > 0.05). The EVOMELA^®^ group had a higher CR rate (73.6% vs. 38.9%) and a lower PR rate (3.8% vs. 13.0%) than the PG-Mel group, indicating superior post-transplantation response outcomes for EVOMELA^®^. Median progression-free survival was 51.4 months (95% confidence interval (CI) 43.5–59.2) in the EVOMELA^®^ group and 49.0 months (95% CI 39.7–58.3) (*p* = 0.115) in the PG-Mel group (HR 0.76, 95% CI 0.49–1.08; *p* = 0.116). Median overall survival was 56.2 months (95% CI 51.3–61.1) in the EVOMELA^®^ group and 57.9 months (95% CI 53.5–62.4) (*p* = 0.007) in the PG-Mel group (HR 0.57, 95% CI 0.37–0.86; *p* = 0.008). The EVOMELA^®^ group had a higher rate of minimal residual disease (MRD) negativity after ASCT (73.6% vs. 48.1%, *p* = 0.007) than the PG-Mel group.

**Conclusion:**

EVOMELA^®^ appears to demonstrate better efficacy and safety compared to PG-Mel; nonetheless, considering the study’s limitations, these observations warrant further rigorous investigation to confirm their validity.

## 1 Introduction

Multiple myeloma (MM) is a heterogeneous malignant disease characterized by the uncontrolled proliferation of terminally differentiated plasma cells in the bone marrow, resulting in the production of non-functional intact monoclonal immunoglobulins or immunoglobulin chains (1, 2). Worldwide, MM accounts for approximately 1% of all cancers and 10–15% of all hematological neoplasms (3). The prevalence and incidence of MM in China are 6.88/100,000 per year and 1.60/100,000 per year, respectively (4). Despite significant advancements in the treatment of MM that have improved patients’ survival over the past 20 years, currently the sequential treatment of induction, high-dose melphalan, autologous stem cell transplantation (ASCT), and maintenance remains the standard of care worldwide for newly diagnosed transplantation-eligible patients (5). The treatment principle of high-dose melphalan (HDM) followed by ASCT is to eliminate the abnormally proliferating cells in the bone marrow by myeloablative high-dose chemotherapy and/or radiotherapy, followed by re-infusing the patient’s own hematopoietic stem cells for restoration of hematopoietic function and immune reconstitution. As an alkylating agent with anti-tumor activity, high-dose melphalan represents the standard myeloablative conditioning regimen prior to ASCT in multiple myeloma (6, 7).

Conventional melphalan (PG-Mel) is a lyophilized formulation requiring the addition of propylene glycol (PG) as a co-solvent to improve its chemical stability and water solubility (8). However, PG-Mel develops impurities within 30 min after reconstitution and remains stable for only 1 h at room temperature. The potency of PG-Mel decreases with further dilution. In addition, propylene glycol has been associated with an array of side effects, including cardiac, neurological, metabolic, and renal toxicity (9, 10). The potential risk of underdose and increased toxicities related to propylene glycol may prevent physicians from using high doses of melphalan for myeloablative conditioning. In recent years, propylene glycol-free melphalan (PGF-Mel, EVOMELA^®^) has been a new formulation of melphalan that incorporates Captisol (sodium sulfobutyl ether *β*-cyclodextrin, a specially modified cyclodextrin) in order to further improve the solubility and stability of melphalan. This new formulation with Captisol^®^ allows a direct reconstitution by saline without the need for propylene glycol, thus eliminating the toxicities of propylene glycol. In addition, EVOMELA^®^ remains stable for approximately 8–10 h after reconstitution at room temperature (11), which permits a prolonged infusion for the myeloablative conditioning that may ensure the full, intended therapeutic dose of IV melphalan (12).

The safety and efficacy of EVOMELA^®^ have been demonstrated in three clinical trials. In a Phase IIa crossover study, this agent was considered bioequivalent to Alkeran (13), while the other study confirmed the safety and efficacy of high-dose PGF-Mel as a myeloablative conditioning regimen in MM patients before ASCT (14). Another research reported that high-dose melphalan at a single dose of 200 mg/m^2^ was safe for myeloablative conditioning (15). However, in clinical practice, it is unknown whether there is a significant safety and efficacy difference between high-dose EVOMELA^®^ and traditional melphalan (PG-Mel) in Chinese patients with MM undergoing ASCT. We conducted a retrospective cohort study by analyzing real-world data to evaluate the differences in safety and efficacy between the two melphalan formulations in clinical practice.

## 2 Methods

### 2.1 Patients and procedures

This was a single-center retrospective study of patients with MM. Approval from the Ethics Committee of Affiliated Beijing Chaoyang Hospital of Capital Medical University in China was obtained prior to conducting the study.

We identified and reviewed the medical records of 107 qualified patients diagnosed with MM and treated with either EVOMELA^®^ or PG-Mel from June 2018 to April 2022 at the Beijing Medical Research Center for Multiple Myeloma, Beijing Chaoyang Hospital, affiliated with Capital Medical University. The key inclusion criteria included patients who had adequate organ function, defined as follows: cardiac function (left ventricular ejection fraction ≥ 50%), hepatic function (total bilirubin ≤1.5 × upper limit of normal (ULN), and serum alanine aminotransferase (ALT)/aspartate aminotransferase (AST) ≤ 2.5 × ULN), renal function (creatinine clearance (CrCl) ≥ 40 mL/min), pulmonary function (diffusing capacity of the lung for carbon monoxide (DLCO) ≥ 60% of predicted value). Additionally, a Barthel index score for activities of daily living (ADL) > 60 was required. The exclusion criteria included patients with a history of allergic reaction to Captisol or compounds similar to PG-free melphalan; history of other malignancies; HIV positivity or uncontrolled intercurrent illness (e.g., known chronic obstructive pulmonary disease with a forced expiratory volume in 1 s (FEV1) < 50% of predicted normal or currently having uncontrolled asthma of any classification, uncontrolled diabetes, unstable angina, and congestive heart failure) that could interfere with study procedures or outcomes.

The patients were categorized into two groups: the EVOMELA^®^ group (53 patients) and the PG-Mel group (54 patients). Stem cell storage and cryopreservation were performed in accordance with the standard operating procedure established by the Department of Hematology at Beijing Chaoyang Hospital for stem cell storage. The median time from autologous hematopoietic stem cell collection to high-dose melphalan preconditioning was 2.3 months. Patients enrolled in the study were administered 200 mg/m^2^ of i.v. melphalan in two doses of EVOMELA^®^ or PG-Mel (100 mg/m^2^ each, with an infusion duration of approximately 30 min) on days −3 and −2 followed by a day of rest before ASCT on day 0. The 2-day dosing regimen (100 mg/m^2^/day) strictly adhered to the FDA-approved prescribing information for EVOMELA^®^ (melphalan for injection), ensuring consistency with established safety and efficacy profiles. There was one exception: one patient with CrCl 43 mL/min (meeting the inclusion threshold of ≥40 mL/min) received reduced-dose PG-Mel 70 mg/m^2^ daily for 2 days due to a relatively lower creatinine clearance rate. In the analysis, patients showed no rise in white blood cell counts following melphalan administration; instead, they demonstrated a gradual decline in white blood cells after medication, ultimately achieving marrow ablation. All our patients who underwent transplant had their bone marrow samples analyzed for minimal residual disease (MRD) both pre-and post-transplantation. All patients provided written informed consent prior to transplantation.

Efficacy and safety data were collected. Continuous safety monitoring was conducted throughout the study period, including up to 30 days following the final administration of the investigational treatment. Adverse events, overall response rate (ORR), myeloablation, neutrophil engraftment, and platelet engraftment were assessed by site investigators as defined below. Adverse events were assessed and graded according to the National Cancer Institute Common Terminology Criteria for Adverse Events (CTCAE, version 5.0) (16). MM response rates were assessed according to the International Myeloma Working Group (IMWG) uniform response criteria before ASCT (17). The International Staging System (ISS) was used to stage patients at the time of diagnosis (18). The fluorescence *in situ* hybridization (FISH) groups were classified according to the 2016 IMWG guidelines into high-risk and standard-risk groups (19).

### 2.2 Statistical analyses

Differences in the frequency of TRAE [ionic disorders (hypokalemia, hypocalcemia, and hypophosphatemia), gastrointestinal reactions (diarrhea, nausea, vomiting, and decreased appetite), and pyrexia], time to myeloablation, time-to-neutrophil engraftment, time to platelet engraftment, the neutrophil nadir (10^9^/L), lymphocyte nadir (10^9^/L), the frequency of platelet transfusions, the average length of a hospitalization stay, and MRD negativity rate were compared between the two groups of patients.

The Kaplan–Meier methodology was used to summarize time-to-event variables e.g., time to neutrophil engraftment, time to platelet engraftment, time to myeloablation, progression-free survival (PFS), overall survival (OS). Kaplan–Meier methodology was used to estimate time-to-event distributions, with stratified log-rank tests, and Cox models (two-sided *α* of 0·05) used for inter-arm comparisons of time-to-event endpoints. The rates of myeloablation, engraftment (neutrophils and platelets), and non-engraftment in the study population were assessed based on the definitions below and were summarized by the proportions of patients meeting each criterion.

Data processing was performed using SPSS 27.0 software. Continuous data following a normal distribution were presented as mean ± standard deviation (SD), and group comparisons were conducted using the two independent samples t-test. Non-normally distributed data were presented as an interquartile range (IQR), and group comparisons were performed using the two independent samples Mann–Whitney U-test. Categorical variable data were expressed as frequency (percentage), and group comparisons were carried out using the chi-square test. A statistically significant difference was defined as a *p*-value of < 0.05.

### 2.3 Definition

Treatment-emergent adverse events (TEAEs) are adverse events that occurred after the first study treatment up to 30 days after the last study treatment. The date of myeloablation is the first of 2 consecutive days in which the cell count met any one of the three criteria: an absolute neutrophil count <0.5 × 10^9^/L, an absolute lymphocyte count<0.1 × 10^9^/L, or an absolute platelet count <20 × 10^9^/L. Time to myeloablation is calculated as “date of myeloablation - date of myeloablative conditioning regimen+1.” Neutrophil engraftment is defined as a sustained drop in blood count to a nadir and then back up again after transfusion of autologous hematopoietic stem cells with a neutrophil count>0.5×10^9^/L for 3 consecutive daily assessments. The date of neutrophil engraftment was the first of 3 consecutive days on which the neutrophil count was>0.5×10^9^/L. Time-to-neutrophil engraftment was calculated as “date of neutrophil engraftment - date of ASCT+1.” Platelet engraftment was defined as a sustained drop in blood count to a minimum and then a rebound, with a platelet count >20 × 10^9/^L for 3 consecutive days, in the absence of a platelet transfusion in the preceding 7 days, after autologous hematopoietic stem cells. The date of platelet graft implantation was the first of 3 consecutive days with a platelet count >20 × 10^9^/L. Time-to-platelet engraftment was calculated as “date of platelet engraftment- date of ASCT+1.” The average length of a hospitalization stay was calculated as “date of discharge—date of hospitalization + 1.” The exploratory endpoint progression-free survival (PFS) was defined as the time from transplantation to the date of either the first observation of progressive disease or the occurrence of death due to any cause, whichever occurred first. The exploratory endpoint, overall survival (OS), was defined as the time from transplantation to the date of death.

## 3 Results

### 3.1 Comparison of clinical characteristics between the two groups

One hundred and seven MM patients underwent ASCT: 53 patients were treated with EVOMELA^®^, while 54 patients were treated with PG-free Mel for conditioning. The two treatment groups were matched with age, sex, pretransplantation response, type of myeloma, ISS stage at diagnosis, Barthel index score of ADL, and the presence of high-risk cytogenetics at diagnosis detected by FISH. Comparison of the baseline data between the two groups demonstrated no statistically significant differences (*p* > 0.05). The details are presented in Table 1.

**Table 1:** Comparison of baseline data between the two groups.

Characteristic	EVOMELA^®^ group (*N* = 53)	PG-Mel group (*N* = 54)	t /χ^2^ value	*P*-value
Age (years), mean ± SD	53.30 ± 6.56	54.43 ± 7.57	−0.820	0.414
Male, n (%)	33 (62.3)	25 (46.3)	2.747	0.097
Pretransplantation response, n (%)			5.522	0.137
Stringent complete response/complete response	21 (39.6)	15 (27.8)		
Very good partial response	17 (32.1)	12 (22.2)		
Partial response	12 (22.6)	23 (42.6)		
Stable disease/progressive disease	3 (5.7)	4 (7.4)		
Type of myeloma, n (%)			4.508	0.212
IgG	25 (47.2)	22 (40.7)		
IgA	7 (13.2)	11 (20.4)		
Light chain	8 (15.1)	14 (25.9)		
Other^a^	13 (24.5)	7 (13.0)		
ISS at diagnosis, n (%)			2.515	0.473
I	13 (24.5)	18 (33.3)		
II	16 (30.2)	10 (18.5)		
III	19 (35.8)	19 (35.2)		
Missing data	5 (9.4)	7 (13.0)		
FISH cytogenetics, n (%)			0.101	0.750
Standard risk	33 (62.3)	32 (59.3)		
High risk	20 (37.7)	22 (40.7)		
Barthel index score of ADL before ASCT, score (%)			0.001	0.977
100	33 (62.3)	36 (66.7)		
61–99	13 (24.5)	14 (25.9)		
Missing data	7 (13.2)	4 (7.4)		

^a^ Other types of myeloma included two patients with the double-clone type of myeloma in the EVOMELA^®^ group and two patients with missing data in the PG-Mel group.

### 3.2 Comparison of the occurrence of adverse events between the two groups of patients

Common adverse events that occurred in patients during treatment included ionic disorders (hypokalemia, hypocalcemia, and hypophosphatemia) [1 case (1.9%) in the EVOMELA^®^ group, 12 cases (22.2%) in the PG-Mel group], nausea [20 cases (37.7%) in the EVOMELA^®^ group, 31 cases (57.4%) in the PG-Mel group], diarrhea [11 cases (20.8%) in the EVOMELA^®^ group, 24 cases (44.4%) in the PG-Mel group], vomiting [3 cases (5.7%) in the EVOMELA^®^ group, 11 cases (20.4%) in the PG-Mel group], decreased appetite [17 cases (32.1%) in the EVOMELA^®^ group, 29 cases (53.7%) in the PG-Mel group], and pyrexia [20 cases (37.7%) in the EVOMELA^®^ group, 24 cases (44.4%) in the PG-Mel group]. Comparing the frequency of the above adverse events between the EVOMELA^®^ group and the PG-Mel group, ionic disorders (hypokalemia, hypocalcemia, and hypophosphatemia), nausea, diarrhea, vomiting, and loss of appetite statistically significantly vary (*p* < 0.05), and pyrexia was not statistically significant (*p* > 0.05), as shown in Table 2.

**Table 2:** Comparison of the frequency of adverse events between the two groups [*n* (%)].

Adverse events	EVOMELA^®^ group (*N* = 53)	PG-Mel group (*N* = 54)	χ^2^ value	*P*-value
All adverse events, n	53	54		
Ionic disorders (hypokalemia, hypocalcemia, hypophosphatemia), n (%)	1 (1.9)	12 (22.2)	10.363	0.001
Vomiting, n (%)	3 (5.7)	11 (20.4)	5.089	0.024
Diarrhea, n (%)	11 (20.8)	24 (44.4)	6.82	0.009
Decreased appetite, n (%)	17 (32.1%)	29 (53.7)	5.105	0.024
Pyrexia, n (%)	20 (37.7%)	24 (44.4)	0.497	0.481
Nausea, n (%)	20 (37.7)	31 (57.4)	4.149	0.042

### 3.3 Comparison of common adverse events during treatment in the safety population between the two groups of patients

No new safety concerns were identified with longer follow-up. Grade 2 or higher adverse events were reported more frequently in the PG-Mel group than in the EVOMELA^®^ group. The most common (in >12% of patients in either group) grade 2 or higher treatment-emergent adverse events were diarrhea (2 [3.8%] patients in the EVOMELA^®^ group vs. 7 [13.0%] patients in the PG-Mel group), as shown in Table 3.

**Table 3:** Comparison of common adverse events during treatment in the safety population between the two groups [n (%)].

Adverse events	EVOMELA^®^ group (*N* = 53)				PG-Mel group (*N* = 54)			
	Any grade	Grade 1	Grade 2	Grade 3	Any grade	Grade 1	Grade 2	Grade 3
All adverse events, n	53				54			
Ionic disorders (hypokalemia, hypocalcemia, hypophosphatemia), n (%)	1 (1.9)	1 (1.9)	0	0	12 (22.2)	10 (18.5)	2 (3.7)	0
Vomiting, n (%)	3 (5.7)	3 (5.7)	0	0	11 (20.4)	10 (18.5)	1 (1.9)	0
Diarrhea, n (%)	11 (20.8)	9 (17.0)	2 (3.8)	0	24 (44.4)	17 (31.5)	6 (11.1)	1 (1.9)
Decreased appetite, n (%)	17 (32.1)	16 (30.2)	1 (1.9)	0	29 (53.7)	27 (50.0)	2 (3.7)	0
Pyrexia, n (%)	20 (37.7)	18 (34.0)	2 (3.8)	0	24 (44.4)	20 (37.0)	3 (5.6)	1 (1.9)
Nausea, n (%)	20 (37.7)	18 (34.0)	2 (3.8)	0	31 (57.4)	27 (50.0)	4 (7.4)	0

### 3.4 Comparison of time to myeloablation and blood cell counts between the two groups of patients

**Figure 1:**
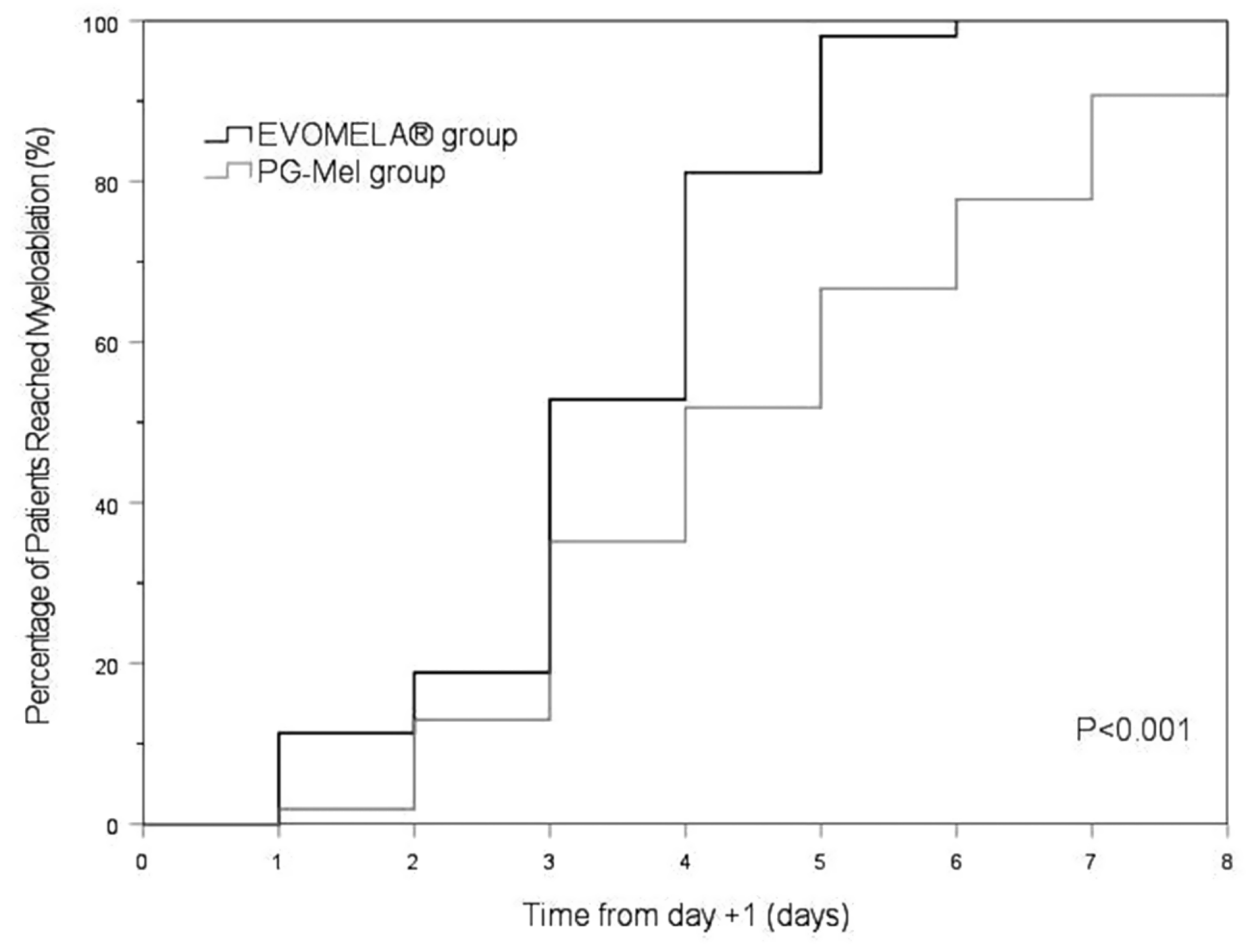
Kaplan–Meier plot of time to myeloablation in both groups.

**Figure 2:**
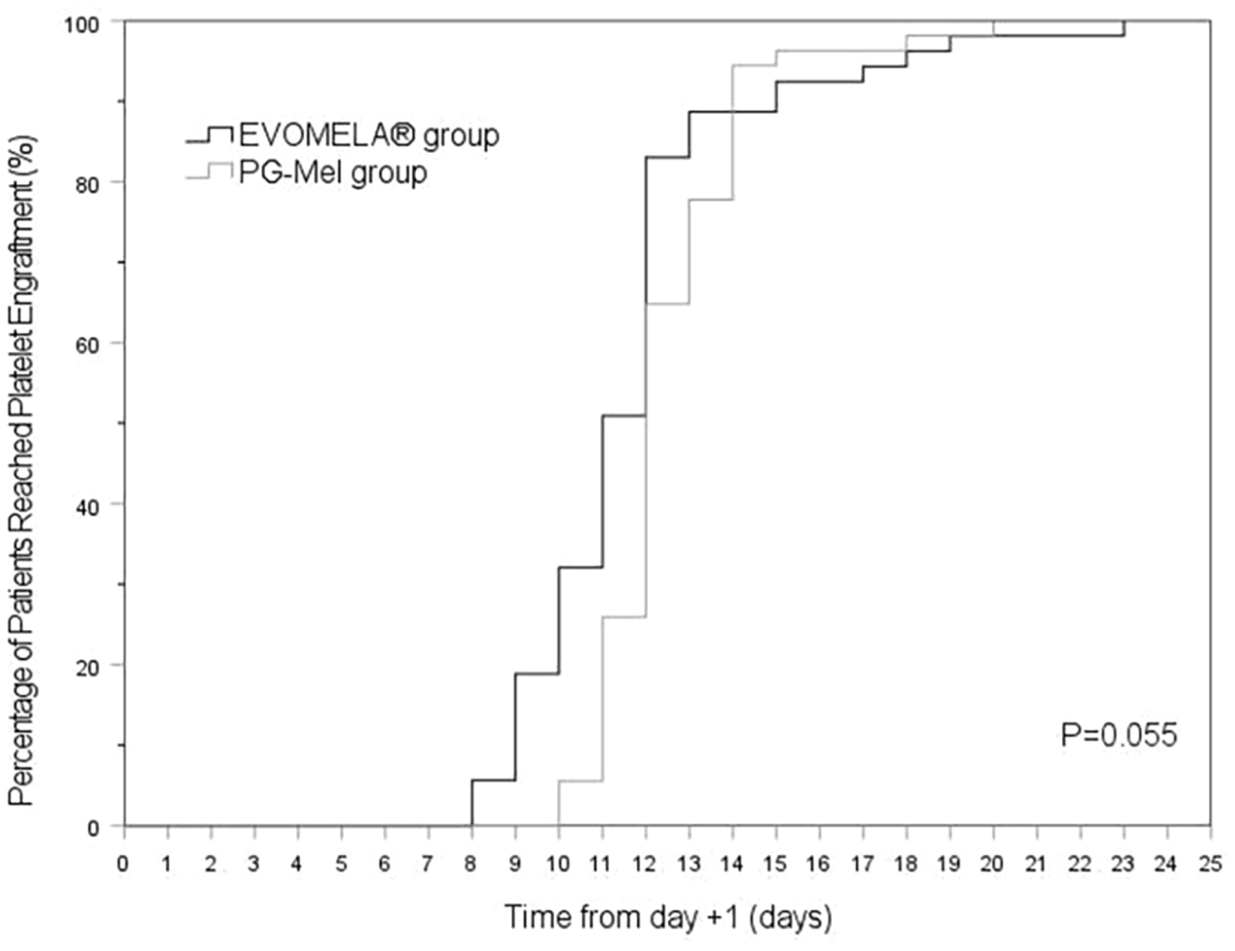
Kaplan–Meier plot of time to platelet engraftment in both groups.

**Figure 3:**
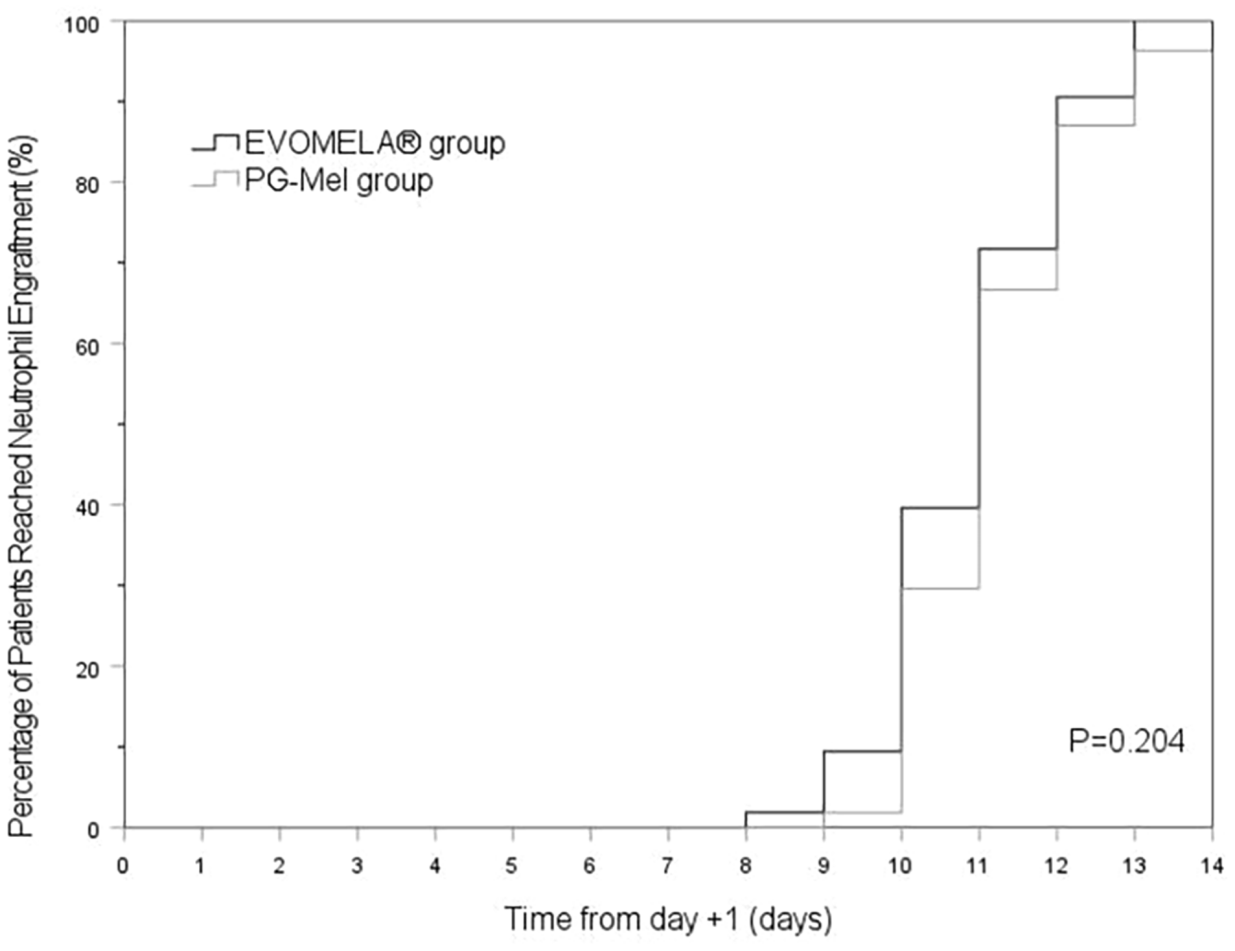
Kaplan–Meier plot of time to neutrophil engraftment in both groups.

The EVOMELA^®^ group exhibited significantly shorter median time to myeloablation (Figure 1), median time to platelet engraftment (Figure 2), and lower neutrophil nadir compared to the PG-Mel group (*p* < 0.05). However, no statistically significant differences were observed between the two groups in terms of median time to neutrophil engraftment (Figure 3), lymphocyte nadir, and the number of platelet transfusions (*p* > 0.05). The detailed results are presented in Table 4.

**Table 4:** Comparison of time to myeloablation and blood cell counts between the two groups of patients.

Parameter	EVOMELA^®^ group (*N* = 53)	PG-Mel group (*N* = 54)	Z value	*P*-value
Time-to-myeloablation (days), median (IQR)	3 (3,4)	4 (3,6)	−3.168	0.002
Time-to-neutrophil engraftment (days), median (IQR)	11 (10,12)	11 (10,12)	−1.221	0.222
Time to platelet engraftment (days), median (IQR)	11 (10,12)	12 (11,13)	−3.134	0.002
Neutrophil nadir (10^9^ /L), median (IQR)	0 (0,0.01)	0.01 (0,0.02)	−2.451	0.014
Lymphocyte nadir (10^9^ /L), median (IQR)	0 (0,0.02)	0.01 (0,0.02)	−1.652	0.099
Total numbers of platelet transfusions (times), median (IQR)	2 (1,2)	2 (1,2)	−0.117	0.907

### 3.5 Comparison of the average length of a hospitalization stay between the two groups of patients

The difference in the average length of a hospitalization stay between the two groups was not statistically significant (*p* > 0.05), as shown in Table 5.

**Table 5:** Comparison of the average length of a hospitalization stay between the two groups.

Parameter	EVOMELA^®^ group (*N* = 53)	PG-Mel group (*N* = 54)	Z value	*P*-value
Average length of a hospitalization stay (days) median (IQR)	21 (19, 23)	21 (19, 22)	−0.417	0.677

### 3.6 Comparison of responses after transplantation between the two groups of patients

The overall response rate (ORR) was 100% in the EVOMELA^®^ group, comprising a complete response (CR) rate of 73.6% (including 0% stringent complete response [sCR]), a very good partial response (VGPR) rate of 22.6%, a partial response (PR) rate of 3.8%, and a stable disease (SD) rate of 0%. In the PG-Mel group, the ORR was 98.2%, with a CR rate of 38.9% (0% sCR), VGPR rate of 46.3%, PR rate of 13.0%, and SD rate of 1.8%. Comparative analysis of post-transplantation responses between the two groups revealed statistically significant differences (*p* < 0.05) (Table 6).

**Table 6:** Comparison of responses after transplantation between the two groups of patients.

Parameter	EVOMELA^®^ group (*N* = 53)	PG-Mel group (*N* = 54)	t /χ^2^ value	*P*-value
Response after transplantation, n (%)			13.996	<0.001
Stringent complete response/complete response	39 (73.6)	21 (38.9)		
Very good partial response	12 (22.6)	25 (46.3)		
Partial response/stable disease	2 (3.8)	8 (14.8)		

### 3.7 Comparison of progression-free survival and overall survival between the two groups of patients

**Figure 4:**
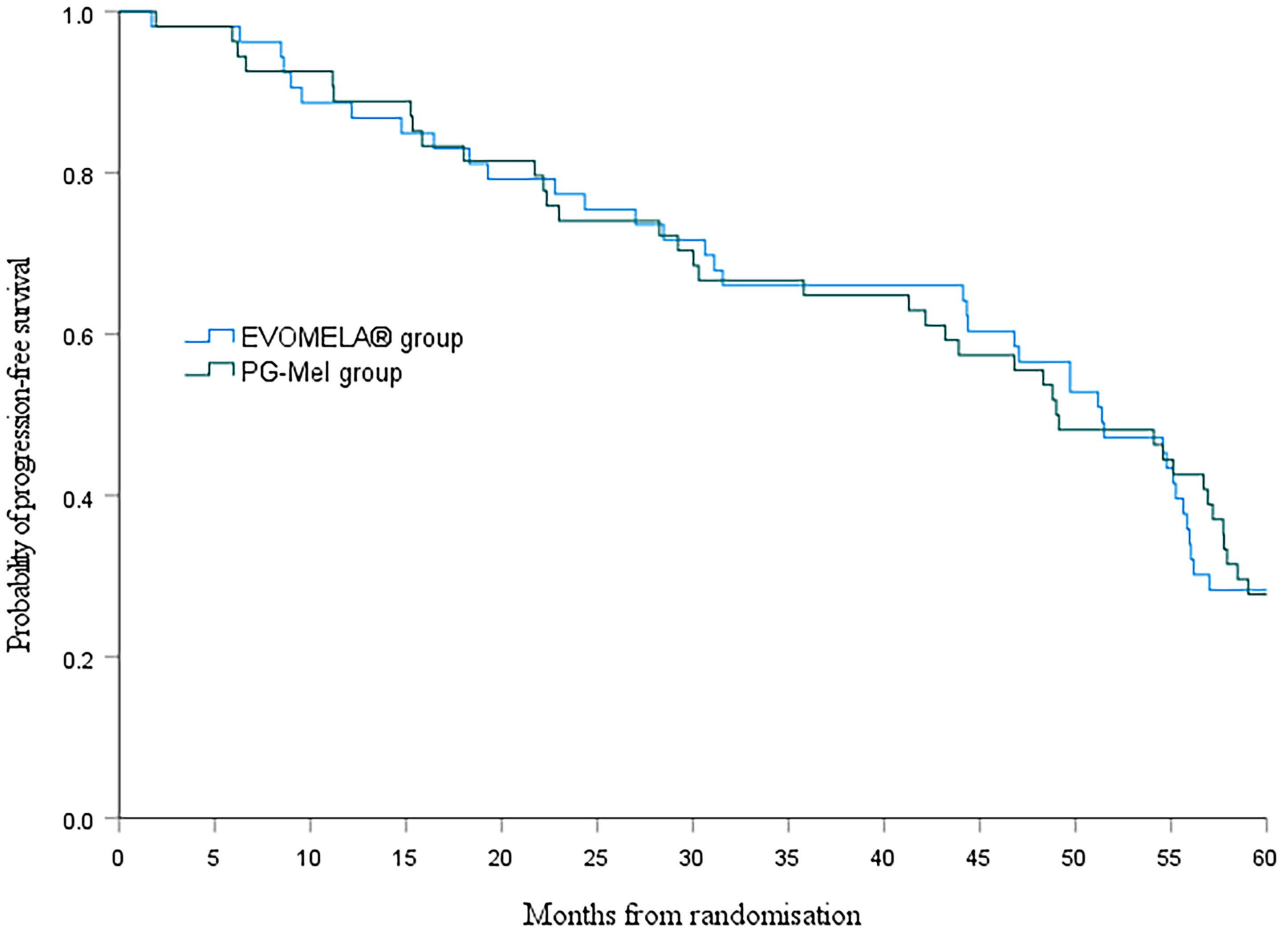
Kaplan–Meier plot of progression-free survival in both groups.

**Figure 5:**
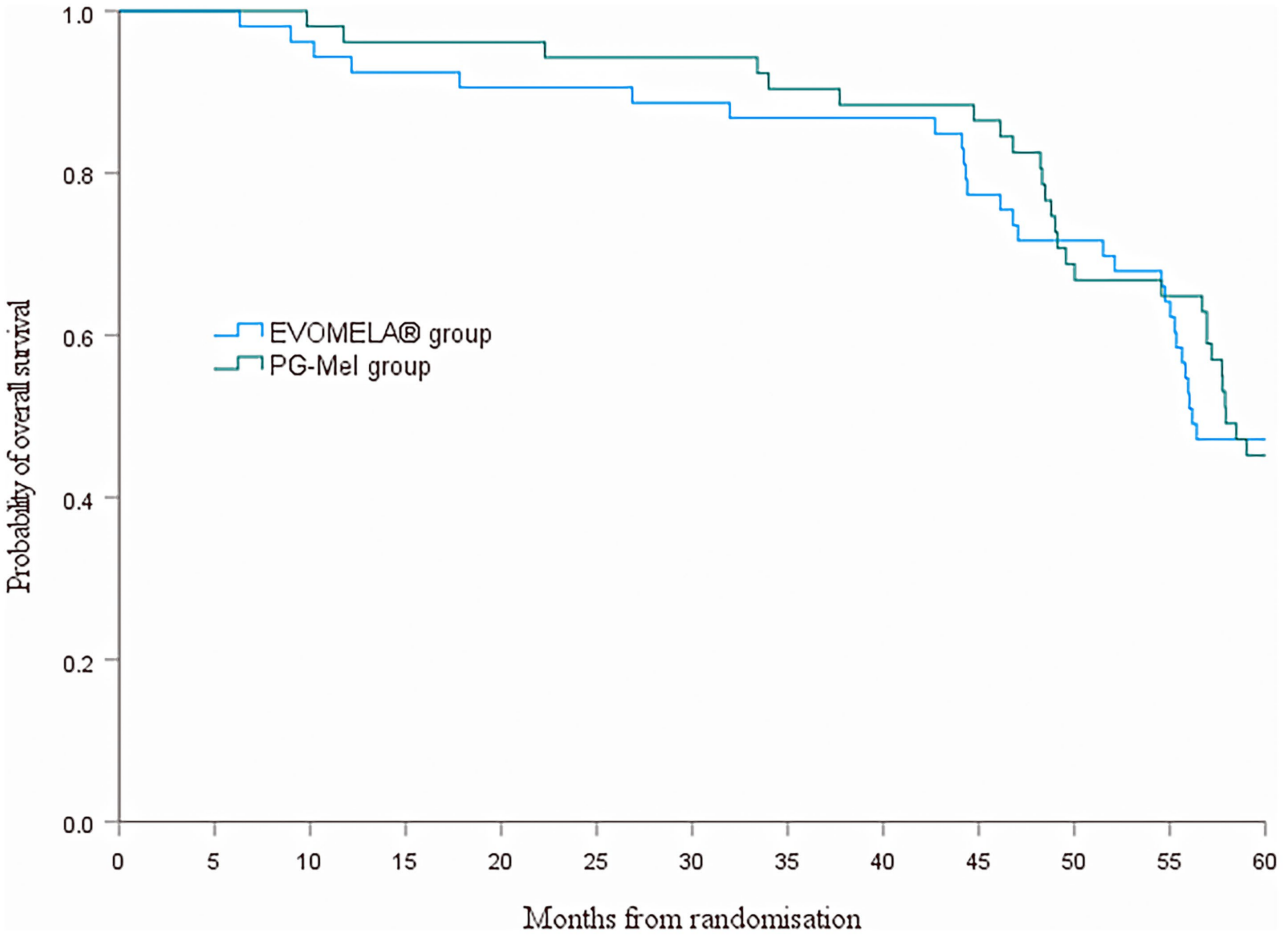
Kaplan–Meier plot of overall survival in both groups.

At the data cutoff for this analysis (22 July 2025), median PFS was 51.4 months (95% confidence interval (CI) 43.5–59.2) in the EVOMELA^®^ group and 49.0 months (95% CI 39.7–58.3) (*p* = 0.115) in the PG-Mel group (HR 0.76, 95% CI 0.49–1.08; *p* = 0.116) (Figure 4). Median OS was 56.2 months (95% CI 51.3–61.1) in the EVOMELA^®^ group and 57.9 months (95% CI 53.5–62.4) (*p* = 0.007) in the PG-Mel group (HR 0.57, 95% CI 0.37–0.86; *p* = 0.008) (Figure 5).

### 3.8 MRD negativity results

Between June 2018 and April 2022, 107 patients who underwent ASCT were tested for MRD status both pre- and post-transplantation (53 patients received EVOMELA^®^ and 54 received PG-Mel). The rate of MRD negativity was similar between the two treatment groups before ASCT: 8/53 (15.1%) in the EVOMELA^®^ group and 10/54 (18.5%) in the PG-Mel group (*p* = 0.636). However, the rate of MRD negativity after ASCT was statistically significant, with 73.6% in the EVOMELA^®^ group versus 48.1% in the PG-Mel group (*p* = 0.007) (Table 7).

**Table 7:** MRD negativity results in both groups.

MRD	EVOMELA^®^ group (*N* = 53)	PG-Mel group (*N* = 54)	t /χ^2^ value	*P*-value
Prior -ASCT, n (%)				
			0.224	0.636
MRD negative	8 (15.1)	10 (18.5)		
MRD positive	45 (84.9)	44 (81.5)		
Post-ASCT, n (%)				
			7.258	0.007
MRD negative	39 (73.6%)	26 (48.1)		
MRD positive	14 (26.4)	28 (51.9)		

## 4 Discussion

High-dose melphalan is the most widely used myeloablative conditioning regimen in clinical practice and is recommended by numerous national and international guidelines. Despite the emergence of various new drugs, high-dose melphalan combined with autologous stem cell transplantation (HDM-ASCT) remains the standard of care for newly diagnosed multiple myeloma patients who are eligible for transplantation and will likely remain so for many years (20). However, the use of propylene glycol as a co-solvent in melphalan formulation in high-dose myeloablative conditioning has presented certain limitations in terms of solubility and stability. In addition, propylene glycol may contribute to adverse effects, such as renal dysfunction. By replacing propylene glycol with Captisol^®^, EVOMELA^®^ overcomes the solubility and stability challenges of PG-Mel, allowing it to directly reconstitute in normal saline and stabilize for a minimum of 8 h at room temperature, ensuring that it can be used in adequate doses for a high-dose melphalan conditioning regimen. In addition, unlike PG-Mel, its stability can be extended up to 24 h under refrigerated conditions, which enhances the convenience and flexibility of clinical dosing while avoiding the potential adverse effects of propylene glycol.

This study aimed to compare the safety and efficacy differences between EVOMELA^®^ and PG-Mel based on real-world data. The safety assessment included evaluating the frequency of common adverse events such as ionic disorders (hypokalemia, hypocalcemia, and hypophosphatemia), nausea, diarrhea, vomiting, loss of appetite, and pyrexia. Efficacy assessment encompassed evaluating the time to myeloablation, time-to-neutrophil engraftment, time-to-platelet engraftment, neutrophil nadir, lymphocyte nadir, number of platelet transfusions, the average length of a hospitalization stay, response after transplantation, and MRD negativity rate both pre- and post-transplantation. The study results are presented below:

In terms of safety, previous studies have shown that the advantages of EVOMELA^®^ over propylene glycol melphalan are more pronounced, including a lower rate of patient re-hospitalization, a lower rate of grade 3–4 infections, a lower risk of arrhythmias, and a lower risk for patients with renal insufficiency (21, 22). The two groups of patients in this study received EVOMELA^®^ and propylene glycol melphalan, respectively, and there were no deaths and no unanticipated serious adverse events observed during the study. Ionic disturbances (hypokalemia, hypocalcemia, and hypophosphatemia) (1.9% vs. 22.2%, *p* = 0.001), nausea (37.7% vs. 57.4%, *p* = 0.042), diarrhea (20.8% vs. 44.4%, *p* = 0.009), vomiting (5.7% vs. 20.4%, *p* = 0.024), and loss of appetite (32.1% vs. 53.7%, *p* = 0.024) were lower than the frequency of the propylene glycol melphalan group, and the difference was statistically significant (*p* < 0.05), while in the frequency of pyrexia (37.7% vs. 44.4%, *p* = 0.481), the EVOMELA^®^ group was lower than the propylene glycol melphalan group, but the difference was not statistically significant (*P*>0.05). Ionic disorders (hypokalemia, hypocalcemia, and hypophosphatemia), nausea, diarrhea, vomiting, and decreased appetite were significantly better in the EVOMELA^®^ group than in the propylene glycol melphalan group. Instances of Grade 2 or higher adverse events were more prevalent in the PG-Mel cohort compared to the EVOMELA^®^ group, with diarrhea being the predominant Grade 2 or higher adverse event. The lower adverse events and less gastrointestinal discomfort caused by pretreatment in the EVOMELA^®^ group are conducive to reducing patients’ physical and psychological pain, improving patients’ compliance, and enabling patients to actively cooperate with physicians to help the pretreatment proceed smoothly, and thus improving the efficiency of the treatment.

In terms of efficacy, it has been shown that EVOMELA^®^ is bioequivalent to PG-Mel, and EVOMELA^®^ has certain advantages over propylene glycol melphalan, mainly in terms of higher post-engraftment ≥VGPR (Very Good Partial Response) rates and AUC values, and shorter time to engraftment (13, 14). Analysis of post-transplantation response between the two groups revealed statistically significant differences (*p* < 0.05). The EVOMELA^®^ group achieved a higher complete response rate of 73.6% compared to the PG-Mel group’s 38.9%. Conversely, the EVOMELA^®^ group had a lower partial response rate of 3.8% than the PG-Mel group’s 13.0%. These results suggest that the EVOMELA^®^ group may exhibit favorable post-transplantation response outcomes relative to the PG-Mel group, though this trend should be interpreted with caution given the study’s constraints.

In this study, the treatment response rate among patients undergoing sequential autologous stem cell transplantation (ASCT) following EVOMELA^®^ pretreatment showed significant improvement, consistent with previous studies. Specifically, the complete response (CR) rate, as assessed by the investigator in a foreign phase IIb trial, increased from 10% pre-ASCT to 31%. In contrast, the CR rate in this study increased more substantially, from 39.6% pre-ASCT to 73.6%, even more significantly than the foreign-registered study (14). Given the study’s limitations, the short-term efficacy outcomes in Chinese patients treated with EVOMELA^®^ followed by ASCT appear to be favorable, though further validation is needed to confirm these results.

In terms of time to myeloablation and blood cell counts, 53 patients in the EVOMELA^®^ group and 54 patients in the PG-Mel group reached the myeloablation criteria, and their neutrophils and platelets were successfully engrafted. The median time to myeloablation in the EVOMELA^®^ group was 3 days, which was significantly shorter than the PG-Mel group’s 4 days (*p* < 0.05). Similarly, the median time to platelet implantation was 11 days for EVOMELA^®^ and 12 days for PG-Mel, with a statistically significant difference (*p* < 0.05) observed, a trend consistent with published retrospective analyses from The Ohio State University (21). Although our data showed a shorter time to platelet engraftment in the EVOMELA^®^ group, there was no difference in the number of platelet transfusions between the two groups. The median time to neutrophil engraftment was 11 days in both groups (*p* > 0.05), a trend consistent with published retrospective analysis from the Brigham and Women’s Hospital, Dana-Farber Cancer Institute, and Mayo Clinic Rochester (23, 24), but not with the Medical College of Wisconsin or The Ohio State University (21, 22). These results may be explained by differences in supportive G-CSF use and alternate melphalan dosing/schedule.

Although comparing absolute values of median PFS between clinical trials should be avoided due to confounding factors such as differences between patient populations, treatment durations, and prior treatment exposure, assessing the relative benefit versus a common comparator is appropriate. Although the PFS benefit observed with EVOMELA^®^ compared with PG-Mel in this study was longer than 2 months, there was no difference in median PFS between the two groups (*p* > 0.05).

In our study, we used the MRD negativity rate as an additional metric to discern variations in efficacy between the two treatment groups. Given its robust prognostic value, MRD negativity has been validated as a clinically significant surrogate biomarker for PFS and OS across all disease states in MM (25). While no disparities in MRD negativity rates were observed between the groups pre-Autologous Stem Cell Transplantation (ASCT), the EVOMELA^®^ group exhibited a notably higher MRD negativity rate of 73.6% post-ASCT compared to the PG-Mel group’s 48.1%. This discrepancy could potentially be attributed to variances in the myeloablative conditioning regimens used.

### 4.1 Limitations

There are also some limitations to this study. First, due to the retrospective study design, some initial symptoms of adverse events may have been underestimated, so the true cases may be higher than those recorded. Second, some of the data in this study, such as genetic data, were missing and may not be sufficient to fully reflect the overall accurate picture. Third, indicators such as remission assessment (the International Myeloma Working Group (IMWG) uniform response criteria) were not included in both groups. Fourth, due to relatively low patient numbers, there was a small sample bias in this study. Furthermore, although the differences in the pre-transplant characteristics of the patients were not statistically significant, there appeared to be trends favoring EVOMELA. These limitations may have influenced the study results and their interpretation. Future studies with larger sample sizes, more extensive data collection, more comprehensive statistical methods, and a more detailed assessment of pre-transplant characteristics are needed to further validate the findings and better understand the potential impact of these factors on treatment outcomes.

## 5 Conclusion

In summary, this study conducted a comprehensive analysis of the differences in safety, efficacy, and other aspects. The results show potential favorable trends for EVOMELA^®^ over PG-Mel in certain efficacy and safety outcomes, which could alleviate physical and psychological burdens on patients and enhance adherence and treatment effectiveness. Nevertheless, it is imperative to recognize the study’s constraints, such as its retrospective nature, limited sample size, and incomplete evaluation of pre-transplant variables. These limitations may impact the interpretability of our comparative results, underscoring the preliminary nature of our findings. Subsequent research endeavors should prioritize larger sample sizes, more extensive data collection, and a meticulous appraisal of pre-transplant characteristics to validate these observations and gain deeper insights into the influence of these factors on treatment outcomes.

## Data Availability

The raw data supporting the conclusions of this article will be made available by the authors, without undue reservation.
